# Continuous wavelet transform and higher-order spectrum: combinatory potentialities in breath sound analysis and electroencephalogram-based pain characterization

**DOI:** 10.1098/rsta.2017.0249

**Published:** 2018-07-09

**Authors:** Leontios J. Hadjileontiadis

**Affiliations:** Department of Electrical and Computer Engineering, Aristotle University of Thessaloniki, 54124 Thessaloniki, Greece Department of Electrical and Computer Engineering, Khalifa University of Science and Technology, PO Box 127788, Abu Dhabi, UAE

**Keywords:** continuous wavelet transform, higher-order spectrum, wavelet bispectrum, breathsounds, electroencephalogram, pain characterization

## Abstract

The combination of the continuous wavelet transform (CWT) with a higher-order spectrum (HOS) merges two worlds into one that conveys information regarding the non-stationarity, non-Gaussianity and nonlinearity of the systems and/or signals under examination. In the current work, the third-order spectrum (TOS), which is used to detect the nonlinearity and deviation from Gaussianity of two types of biomedical signals, that is, wheezes and electroencephalogram (EEG), is combined with the CWT to offer a time–scale representation of the examined signals. As a result, a CWT/TOS field is formed and a time axis is introduced, creating a time–bifrequency domain, which provides a new means for wheeze nonlinear analysis and dynamic EEG-based pain characterization. A detailed description and examples are provided and discussed to showcase the combinatory potential of CWT/TOS in the field of advanced signal processing.

This article is part of the theme issue ‘Redundancy rules: the continuous wavelet transform comes of age’.

## 1. Introduction

Signal characterization under the existence of stochastic noise is mostly facilitated by assuming that either the signal or the noise or both signal and noise are Gaussian processes. Further, even when non-Gaussianity is considered, the process is often assumed to be a linear process or a simple martingale (a sequence of random variables for which, at a particular time, the expectation of the next value in the sequence is equal to the present observed value, given the knowledge of all prior observed values) derived from a Gaussian process, i.e. via simple stochastic differential equations [1]. Nevertheless, many real-life processes (e.g. sonar, radar, biomedical and economic) do not fall into the Gaussian and/or linear assumption. Moreover, the existence of non-stationarity alters the statistical characteristics of the process with time, posing an additional factor to be considered in signal analysis. In fact, in most natural systems, the properties of interacting oscillatory systems are not constant, as there is an evolution or fluctuation in time, altering the mutual interaction among subsystems, their frequencies and amplitudes. In addition, the temporary occurrence of frequency and phase coupling, with varying strengths, exposes time-dependent nonlinear interactions between a pair of individual oscillators.

It is apparent, therefore, that, in a time-series setting, nonlinearity, non-Gaussianity and non-stationarity cannot be attained solely via the examination of the spectrum. Identification of nonlinearities in the process, however, could provide an alternative feature space that enhances further their value in the examined problem. This involves the use of higher-order statistics/spectra (HOS) [2], which, unlike the power (second-order) spectrum, preserve the phase information of the signal and could be used to detect the nonlinearity and deviation from Gaussianity of the analysed signals. When combined with the continuous wavelet transform (CWT) [3], which offers a time–scale representation of a signal, a time axis is introduced, assisting the dynamic identification of nonlinearities across the duration of the examined signal. In this way, nonlinearities of signals are revealed in the time–bifrequency domain, also taking into account their non-stationarity.

The combination of the CWT with the Fourier-based third-order spectrum (TOS), namely bispectrum (BS), results in the CWT/TOS, or simply wavelet bispectrum (WBS). The Fourier-based bispectral analysis involves third-order statistics and is used to analyse non-Gaussian signals, to obtain phase information, to suppress Gaussian noise of unknown spectral form, and to detect and characterize signal nonlinearities [2]. The generalization of BS based on Fourier transform to wavelets can be seen as a generalization of the Fourier analysis [4] by adding time resolution (in a more fundamental way than is permitted by the short-time Fourier transform (STFT)). Using bispectral analysis to obtain the time-dependent biphase/biamplitude estimate based on STFT means using a window of constant length. The optimal window length depends, however, on the frequency being studied. The effective length of the window used for each frequency can be varied by applying the CWT. With broader frequency content where natural frequencies lie, the STFT is not sufficient for good time and phase/frequency localization and the CWT needs to be applied [5]. Using WBS, intermittent phase couplings can be detected, whereas Fourier-based BS averages out most of the time relevant information. If there is a brief episode of quadratic phase coupling (QPC), the Fourier-based BS method cannot detect it from the signal due to the large time window used. On the contrary, the WBS will detect it, down to a certain minimum duration; thus the WBS allows intermittent couplings to be detected [4,5]. In fact, WBS represents a significant improvement in the time resolution of the BS.

To date there has been some notable uses of WBS, yet not at the level of the BS alone and even less when compared to the prolific use of the CWT. However, there are some key areas where WBS analysis has made some clear inroads. In particular, WBS has been applied in problems of machine monitoring [6,7] and fault diagnosis [8,9], wind and wave analysis [1,10,11], filter optimization [12], emitter recognition [13], and health-related areas, such as neuroscience [14], mental stress detection [15], wheeze analysis [16,17] and electroencephalogram (EEG)-based tonic cold pain characterization [18]. The main thrust of this paper is to exemplify and discuss the combinatory potential of WBS in the analysis of non-Gaussian, nonlinear and non-stationary processes, taking as paradigms the WBS-based analysis of wheezes [17] and EEG-based tonic cold pain characterization [18].

The paper is outlined as follows. Initially, the definitions of the CWT/TOS-related parameters are provided. Next, selected case studies are presented, including the underlying rationale for each one, the construction of the CWT/TOS-based feature set, the data characteristics, indicative results and relevant discussion. The benefits of the combination of CWT with TOS are made clear. These case studies are summarized in the concluding remarks made at the end of the paper.

## 2. Definition of continuous wavelet transform/third-order spectrum-related parameters

### (a) Continuous wavelet transform (CWT)

The CWT of a signal *x*(*t*)∈*L*^2^(***R***) is defined as [3]
2.1
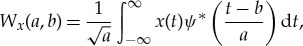

where *x*(*t*) is the signal in the time domain, * denotes the complex conjugate and *ψ*(*t*) is the mother wavelet scaled by a factor *a*, *a* > 0, and dilated by a factor *b*. In the CWT, the time and scale parameters (*a*, *b*) are continuous.

In many cases, the complex Morlet wavelet is preferred as the mother wavelet *ψ*(*t*) in (2.1), given by Addison [3]:
2.2
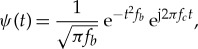

where *f*_*b*_ is a bandwidth parameter and *f*_*c*_ is the wavelet centre frequency. In fact, the complex Morlet wavelet is a Gaussian-windowed complex sinusoid; hence, its second-order exponential decay results in optimal time localization during the wavelet transform. Additionally, the complex Morlet wavelet function provides information about both amplitude and phase, and it is better adapted for capturing coherence between harmonic frequencies [3].

### (b) Third-order spectrum (TOS): bispectrum (BS)

The BS *B*_*x*_(*ω*_1_, *ω*_2_) of a process {*x*(*k*)} is defined as [2]
2.3
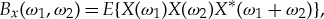

where *E* is the expectation value, *X*(*ω*_*i*_), *i* = 1, 2, is the complex Fourier coefficient of the process {*x*(*k*)} at frequencies *ω*_*i*_ and X*(*ω*_*i*_) is its complex conjugate.

### (c) Third-order coherence (TOC): bicoherence (BC)

The bicoherence (BC) *b*_*x*_(*ω*_1_, *ω*_2_) of a process {*x*(*k*)} is defined as the normalized BS [2], i.e.
2.4
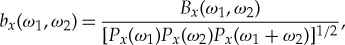

where *P*(*ω*_*i*_), *i* = 1, 2, is the power spectrum of the process {*x*(*k*)} at frequencies *ω*_*i*_.

### (d) Wavelet bispectrum (WBS)

WBS combines CWT with bispectral analysis and, in an analogy to the definition of the BS in Fourier terms (see (2.3)) of a signal *x*(*t*)∈*L*^2^(***R***), it could be defined as [5]
2.5
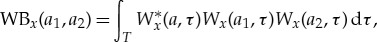


where the integration is performed over a finite time interval *T*:*τ*_0_ ≤ *τ* ≤ *τ*_1_, and *a*, *a*_1_, *a*_2_ satisfy the following rule:
2.6
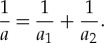


WBS actually expresses the amount of QPC in the interval *T*, which occurs between wavelet components of scale lengths *a*_1_, *a*_2_ and *a* of *x*(*t*), such that the sum rule of (2.6) is satisfied. Taking into consideration the interpretation of the scales as inverse frequencies, i.e. *ω* = 2*π*/*a* or *f* = 1/*a*, the nonlinear coupling reflected in the WBS can be transferred between wavelets of frequencies that satisfy *ω* = *ω*_1_ + *ω*_2_, within the frequency resolution. Owing to the symmetries in the definition and the limitation set by the Nyquist sampling frequency *ω*_s_, the estimation of WBS in the whole bifrequency plane can be based on its values in the principal region Δ:{0 ≤ *ω*_1_, 0 ≤ *ω*_2_ ≤ *ω*_1_, *ω*_1_ + *ω*_2_ ≤ *ω*_s_}.

### (e) Wavelet bicoherence (WBC)/summed WBC (SWBC)

The notion of Fourier-based BC is extended to the CWT/TOS space via the definition of the wavelet bicoherence (WBC), as the normalized WBS [4,6], i.e.
2.7
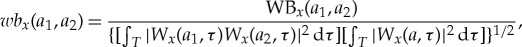

with its magnitude, |*wb*_*x*_(*a*_1_, *a*_2_)|, attaining values between 0 and 1, corresponding to absence (0 value) and strong existence (1 value) of QPC, respectively.

The summed wavelet bicoherence (SWBC) is defined as
2.8
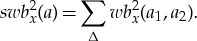

Note that both (2.7) and (2.8) could equivalently be expressed in the bifrequency domain.

### (f) Instantaneous wavelet bispectrum (IWBS)

The involvement of CWT in the bispectral domain provides dynamic monitoring of the evolution of QPC over time. Consequently, the instantaneous wavelet bispectrum (IWBS) can be defined as a time-dependent complex quantity, i.e.
2.9
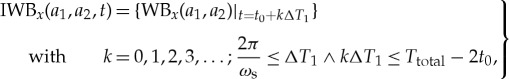

where *T*_total_ is the total time duration of the analysed signal *x*(*t*), *t*_0_ = *T*/2 expresses the time instant to which the estimated parameter corresponds (middle of window) and parameter Δ*T*_1_ controls the time resolution.

### (g) Instantaneous wavelet biamplitude (IWBSAmp) and biphase (IWBSPh)

Rewriting (2.9) in the form of
2.10
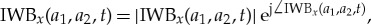

the instantaneous wavelet biamplitude (IWBSAmp) *A*_*x*_(*a*_1_, *a*_2_, *t*) and instantaneous wavelet biphase (IWBSPh) *ϕ*_*x*_(*a*_1_, *a*_2_, *t*) are given by
2.11
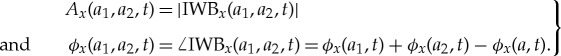


### (h) Instantaneous wavelet bicoherence (IWBC)

In a similar manner to the IWBS, the instantaneous wavelet bicoherence (IWBC) is defined as
2.12
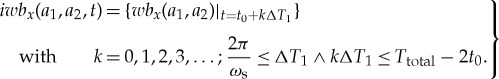


### (i) Instantaneous wavelet bicoherence amplitude (IWBCAmp) and bicophase (IWBCPh)

Equation (2.12) can be expressed as
2.13
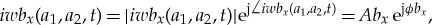

where *Ab*_*x*_ is the instantaneous wavelet bicoherence amplitude (IWBCAmp) and *ϕb*_*x*_ is the instantaneous wavelet bicoherence phase (IWBCPh). It is apparent that, when using IWBS and IWBC, the evolution of the nonlinearities across time can be represented, within a time resolution controlled by the selection of the Δ*T*_1_ value.

In the section that follows, two case studies are presented that explore the potential of CWT/TOS-based features in addressing two health-related problems, i.e. wheeze analysis and EEG-based tonic pain characterization.

## 3. Case studies

### (a) Wheeze analysis

#### (i) Problem and underlying rationale.

Wheezes constitute one of the most commonly met types among abnormal breath sounds, because they are strongly related to patients with obstructive airways diseases, such as asthma and chronic obstructive pulmonary disease (COPD) [19]. Wheezes, exhibiting time duration greater than 150 ms [20], are considered to be continuous adventitious lung sounds. The musical character of wheezes is reflected in their time-domain waveform as a mixture of sinusoidals, leading to the appearance of distinct peaks in the spectrum (greater than 100 Hz) [20]. Wheezes are characterized as adventitious breath sounds, because they are superimposed on normal breath sounds, during either inspiration or expiration, and exhibit varying duration, intensity, frequency content and number of harmonics. The number of wheezes and their distribution across the chest are related to the distribution of the pathology, whereas their complexity, i.e. whether they are monophonic or polyphonic, may suggest a specific disease. A monophonic wheeze consists of a single note or several notes starting and ending at different times. Polyphonic wheezes, however, are made up of several dissonant tones starting and ending simultaneously. Multiple monophonic wheezes which occur simultaneously are a typical sign of asthma. Polyphonic wheezes occur when there is a relatively fixed compression in multiple central bronchi simultaneously and are usually found in COPD patients [21]. To this end, analysis of the harmonic interaction of wheezes becomes important, because their musicality produces distinct spectral peaks that could embed nonlinear characteristics related to the underlying pathology, expressed in their phase relation through QPC.

Through previous studies, wheezes have been characterized as the acoustic manifestation of airways obstruction [22]. Wheeze monitoring has been used for the assessment of nocturnal asthma and response to therapy [23] and wheeze characteristics have been used to rate the severity of asthma, as indicators of airway obstruction in infants, or as classification features in epidemiologic surveys [24]. Although significant scientific effort has addressed the problem of wheeze detection combined with power spectral analysis, a limited number of works have dealt with the nonlinear interactions of their harmonic content. As the nonlinear harmonic peak interaction is affected by changes in the functionality of the lungs due to airways obstruction, identification of such nonlinearities in wheezes could provide an alternative feature space that enhances further their diagnostic value. Some indicative works in this direction include bicoherence-based [25] and phase space analysis [26].

The mathematical background presented in the previous section sets the analysis domain of wheezes as explored in [17]. For the quantification of the information derived from the wheeze analysis in this domain, a set of features was defined. As the WBS/bicoherence analysis incorporates time and bifrequency characteristics, the introduced features are structured in two distinct ways, i.e. (a) those capturing the bifrequency characteristics of the wheezing signal and (b) those affected by the evolution of the wheezing signal over time. Considering that all such features are defined in the time–bifrequency domain, they relate to the nonlinear and non-stationary characteristics of wheezes reflected in the QPC of their distinct harmonic peaks and its evolution over time.

#### (ii) Continuous wavelet transform/third-order spectrum-based features.

In [17], the wheezing signal was dealt with, first, as a single entity (i.e. *T* = *T*_total_ in (2.9) and (2.12)). In this way, the morphology of the derived WBS/WBC could offer information about the existence of such bifrequencies where peaks provide evidence of possible frequency and phase interactions. These peaks are denoted as global peaks (GPs) and their characteristics set the bifrequency-related category of the proposed features. Next, a detailed perspective of wheeze WBS/WBC-based analysis was adopted, considering windowed overlapping sections of the wheezing signal (controlled by the selection of Δ*T*_1_), incorporating IWBS/IWBC analysis. Again, the morphology of the derived IWBS/IWBC provided information about bifrequencies where peaks provide evidence of possible frequency and phase interactions produced by the windowed wheezing signal. These peaks are denoted as local peaks (LPs) and their characteristics set the time–bifrequency-related category of the features used. In addition, as LPs provide a microscopic resolution of the wheezing signal in the time–bifrequency domain, they could be used to form a nonlinearity index (NLI) to express the way the nonlinearity is distributed across the wheezing signal, taking into account that the nonlinearity level of the wheeze included within the *t* interval is characterized as I, II, III, IV and V levels, when the estimated 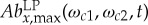 value lies between [0 − 0.2], [0.2 − 0.4], [0.4 − 0.6], [0.6 − 0.8] and [0.8 − 1], respectively, and (*ω*_*c*1_, *ω*_*c*2_, *t*) corresponds to the position of maximum value. The NLI is defined as the ratio of the duration of time intervals characterized as level *j* to the total duration of the signal, i.e.
3.1
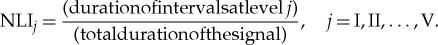


When referring to the GP value, it is considered to be
3.2
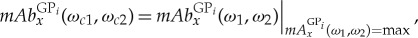

with *c*^*i*^ = (*ω*_*c*1_, *ω*_*c*2_)^*i*^, *i* = 1, 2, …, *l*, being the position of the maximum value of peak *i* and *l* is the number of global peaks. Considering the contour *S* of GP_*i*_ and *s*^*j*^ = (*ω*_s1_, *ω*_s2_)^*j*∈*S*^, *j* = 1, 2, …, *m*, where *m* is the number of points of the contour *S* of GP_*i*_, the Euclidean distance of *s*^*j*^ from *c*^*i*^ can be defined as *D*^GP_*i*_^ = dist(*s*^*j*^, *c*^*i*^). The GP_*i*_ contour mean distance and standard deviation distance are denoted as 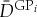 and std(*D*^GP_*i*_^), respectively.

The features that were considered in [17] were: 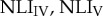, 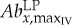, 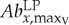, 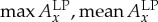, 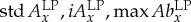, 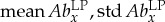, 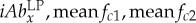, 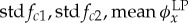, 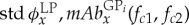, *f*_*c*1_, *f*_*c*2_, 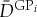 and std(*D*^GP_*i*_^).

#### (iii) Experimental dataset—implementation.

For the CWT/TOS wheeze analysis, 393 wheezes were used, derived from 10 COPD and 11 asthma patients (10 male and 11 female), which were drawn from the MaRS database (Philipps University of Marburg, Germany) [27]. Respiratory acoustic signals were recorded in a semi-quiet clinical laboratory, from five electret condenser microphones (ECM-77B, Sony, Inc., Tokyo, Japan) applied over the trachea, right and left axillae and right and left posterior bases of the lungs. Airflow signals were recorded simultaneously, using a pneumotachograph. The patients breathed through a mouthpiece with a nose-clip and were asked to keep a maximum flow of 1.5 l s^−1^ through visual feedback. Signal conditioning, i.e. amplification and bandpass filtration (60–2100 Hz at 48 dB/oct, Butterworth), was performed prior to analogue-to-digital conversion with a 12-bit resolution at a sampling frequency of *f*_s_ = 5512 Hz. Further parameter setting details can be found in [17].

#### (iv) Indicative results and discussion.

**Figure 1.**
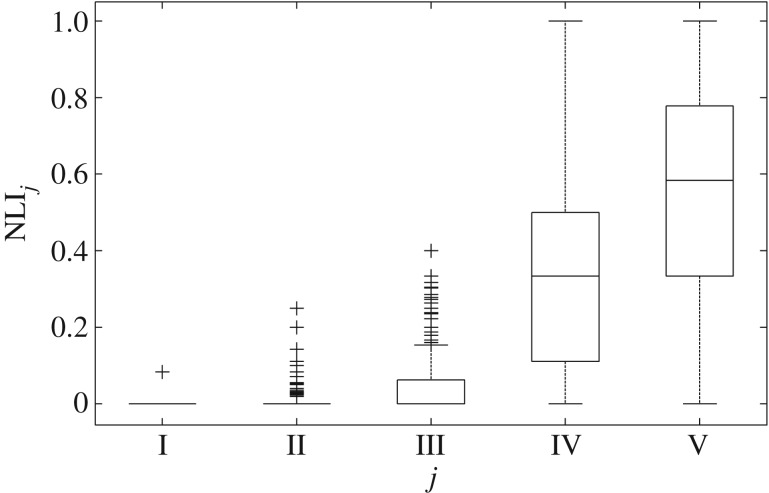
Distribution of the NLI_*j*_, *j* = I, II, …, V, values (adapted from Taplidou & Hadjileontiadis [17]).

**Figure 2.**
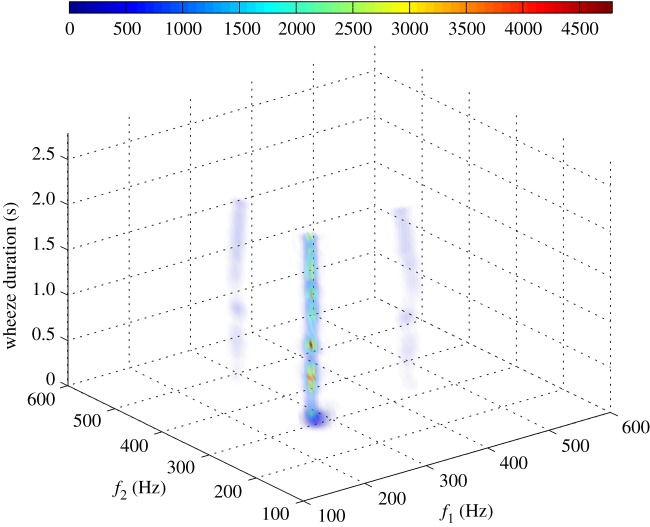
Evolution of the LPs over time through the evolution of the IWBSAmp (adapted from Taplidou & Hadjileontiadis [17]).

**Figure 3.**
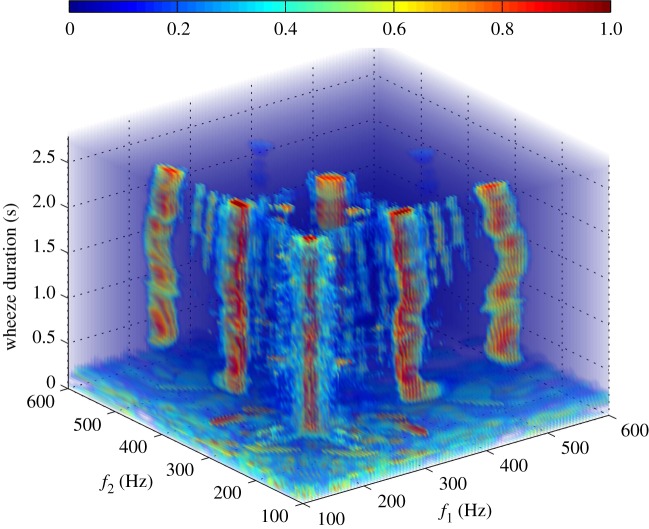
Evolution of the LPs over time through the evolution the IWBCAmp (adapted from Taplidou & Hadjileontiadis [17]).

The boxplot of figure 1 depicts the distribution of the NLI_*j*_, *j* = I, II, …, V, across the dataset. From this figure, it is apparent that high QPC values (levels IV and V) appear across the longest part of the wheeze (median NLI_IV_ value 0.385; median NLI_V_ value 0.544). Furthermore, figures 2 and 3 illustrate how the estimated LPs, corresponding to the nonlinear interaction of the harmonic content of wheezes, evolve over time, via the evolution of the estimated IWBSAmp (figure 2) and IWBCAmp (figure 3). In this way, a visualization of the nonlinear activity across the evolution of the breathing sound is feasible, identifying the time sections of the signal that contribute the most to the nonlinear activity. From figure 2, it can be seen that three main peaks appear across the wheezing signal. The peak due to the quadratic phase auto-coupling of the frequency component at 160 Hz exhibits an increase around 0.5–1.0 s. Nevertheless, as figure 2 shows, a basic level of the nonlinear activity related to the bifrequency pair of (160, 160) Hz is initiated and sustained for the whole duration of the wheeze (0.5–2.75 s).

The evolution of the other bifrequency pair of (160, 320) Hz, however, is more affected by the morphological change of the wheeze signal, showing some degree of discontinuity. The latter is more evident in figure 3, via the evolution of the LPs drawn from the corresponding IWBCAmp. The variability in the intensity and location at the (*f*_1_, *f*_2_)-plane of the quadratic phase (auto)coupling of the bifrequency pairs (160, 160) Hz, (160, 320) Hz, (160, 480) Hz and (320, 320) Hz across the breathing signal displayed in figure 3 reveals the value of the incorporation of the wavelet transform within the higher-order spectral analysis. Clearly, the visualization of figures 2 and 3 justify the way spectral components of the analysed wheeze are transferred into the bifrequency domain, revealing there their nonlinear interaction, yet sustaining their time-varying characteristics. Such valuable information can be used for a potential differentiation between the underlying pathologies [17].

Looking from a methodological point of view, the feature set used in [17] was based on the bispectral analysis of the wheezing signals combined with the wavelet transform. This CWT/TOS-based analysis represents a significant improvement in the time resolution of the BS. This clearly justifies the introduction of the general (GPs) and detailed (LPs) perspectives in the proposed analysis along with the evolution of the LPs over time. From a practical point of view, the introduction of the proposed feature set pinpoints the diagnostic information of wheezes hidden within the nonlinear interaction of their harmonic elements and the way it evolves over time.

In general, the proposed wavelet bispectral analysis provided a novel tool for studying the nature of coupling between two or more nonlinear oscillators whose basic frequencies change considerably in time. From a statistical analysis between the selected features, the diagnostic information that differentiates between COPD and asthma is spread across the feature set according to the grouping standing point (i.e. breathing phase, wheeze type). This shows that the proposed feature set could be applied and adapted accordingly to various analysis scenarios of wheezes. Combined with the development of a classifier based on the proposed feature set, this could further enhance the computerized analysis of wheezes.

### (b) Electroencephalogram-based tonic cold pain characterization

#### (i) Problem and underlying rationale.

Objective pain characterization is essential and has long been desired for clinical pain assessment and management, as subjective perception of the feeling of pain is difficult to quantify [28]. Towards such endeavour, different approaches have been explored so far, with the most commonly one the use of pain intensity measures, including visual analogue scales, numerical rating scales (NRSs) and verbal rating scales [29]. Nevertheless, they are characterized by intrinsic limitations, as they require human intervention (e.g. by physicians or carers) for the patient to perform the rating in an interview-like manner. Most importantly, though, they do not capture a time-dependent dynamic change in pain perception; hence, they can only reflect an integrated perception of pain, related to the accumulated pain experiences. The high temporal resolution of the EEG may provide a solution, as efficient analysis of related dynamic changes in brain activity in the EEG data may effectively reflect the dynamic changes of pain perception.

So far, the tonic cold pain-related EEG signal has been analysed in the frequency domain, focusing mainly on the power spectrum peaks and/or coherence values at specific EEG frequency bands [30,31]. These approaches, with the exception of Chang *et al.* [32], however, neglect the time variation of the pain-related EEG characteristics, as their features rely solely on the frequency content. None of the related works deal with the nonlinear interactions of the harmonic content of the EEG components. The latter is of great importance, because distinct EEG spectral peaks could embed nonlinear characteristics related to the underlying pain perception, expressed in their phase relation through QPC. In this vein, identification of such nonlinearities in the EEG data could provide an alternative feature space that enhances the tonic cold pain characterization problem. This is explored in [18], shifting the focus of analysis at the CWT/TOS domain, introducing it as a potential field for the formation of quantitative features that could dynamically reflect the nonlinear behaviour of EEG signals and be related to tonic cold pain characterization.

#### (ii) Continuous wavelet transform/third-order spectrum-based features—classifiers.

The approach in [18] for tonic cold pain characterization was built on the exploration of three pain characterization scenarios (PCSs), as follows. (a) PCS#1-Relax versus Pain: identification of the two different states of the subject, i.e. when s/he is relaxed or in pain, from an average perspective. (b) PCS#2-Relax to Pain: identification of the transition from relax to pain (mild and severe) state of the subject, for a dynamic monitoring of the severity of the pain perception with time. (c) PCS#3-Subject's Pain Endurance: identification of each subject's endurance to pain, considering the subjectivity in pain perception. To materialize these scenarios, EEG CWT/TOS-based feature sets and different classification processes were adopted and explored.

In particular, for the PCS#1, the amplitude and phase of WBS (2.5), i.e. WBSAmp and WBSPh, SWBC (2.8) and the number of WBSAmp Local Maxima (WBSAmpLM#) were used. For the latter, the WBSAmp was initially thresholded to its fourth quartile, resulting in a number of local maxima that actually expresses the multiplicity of the main QPC pairs in the bifrequency domain. As the PCS#2 tackles the dynamic transition from relax to pain state, the corresponding feature set was constructed by the IWBSAmp and/or IWBSPh parameters (see (2.11)). Finally, for the PCS#3, the pain endurance feature (PE) was used, defined as the estimated pain tolerance time *T*_tol_ normalized to the *T*_P_ = (*t*_end_ − *t*_click1_), i.e.
3.3
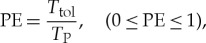

where
3.4
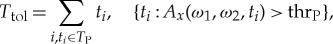

and thr_P_ is a statistically defined threshold (see [18]). The *t*_click1_ and *t*_end_ time instants correspond to the moments when the subject felt the tiny uncomfortable feeling during the pain induced experimental phase, denoting the end of relax state and the beginning of the pain state, and when the pain became unbearable and the experimental trial was terminated, respectively. As the proposed approach was based on the EEG data analysis, their frequency characteristics were taken into consideration for the estimation of the corresponding features. Thus, the average values of the aforementioned features were separately estimated for each of the main frequency bands, i.e. *delta* (1–4 Hz), *theta* (4–8 Hz), *alpha* (8–13 Hz), *beta* (13–30 Hz) and *gamma* (30–49 Hz), transferred to the principal area of the bifrequency domain (Δ).

In addition, the concept of referencing was used for the handling of features, considering the cases of no referencing (NoR) and with referencing state (RS), in which each of the above described features was reshaped to a new *F* form, computed as *F* = (*P* − *R*)/*R*, where *P* represents the quantity (feature) estimated during the pain state period and *R* represents the quantity (feature) estimated during the relax state, used as a RS. Finally, any asymmetric activations that might occur in the brain during the pain state were considered by subtracting (differential asymmetry (DAs)) or dividing (rational asymmetry (RAs)) the estimated features *F* for each of the seven symmetric channel pairs per brain hemisphere, under the EEG 10/20 topology [18].

The different forms of the constructed feature vector were subjected to a 10-fold cross-validation classification procedure, employing quadratic discriminant analysis (QDA) [33], Mahalanobis (MAH) [34], *k*-nearest neighbours (k-NN) [35], and support vector machines (SVM) [36].

#### (iii) Experimental dataset—implementation.

For the experiments, 17 right-handed healthy volunteers (nine males and eight females) of 23.22 ± 1.72 years old were used. Each subject participated with eyes open in a tonic pain stimulation condition, holding with his/her dominant hand a 0.5 litre plastic bottle with iced water (−1°C ± 0.5°C) and simultaneously mouse-click (click0) with his/her non-dominant hand to initiate the experimental trial, to express initial beginning of pain (click1) and the end of pain tolerance (end of experiment trial). The subject was then asked to rate the perceived pain intensity via a NRS, ranging from 0 (no pain) to 10 (maximum pain tolerable). In this vein, s/he was instructed to reflect in his/her NRS value the overall perception of the pain intensity across the whole experiment and not just the one at its end, where the pain is always maximized. The experiment was repeated three more times (four trials in total) with a reset-like procedure in between, lasting more than 5 minutes. EEG recordings (time stamped with the tonic pain stimulation condition events via the subjects' mouse-clicking) were conducted using the Emotiv EPOC 14-channel EEG wireless recording headset (Emotiv Systems Inc., San Francisco, CA) at a sampling frequency of 2048 Hz, and subsequently were filtered in the frequency range of 1 − 49 Hz. Moreover, the EEG data were segmented at *T* = 1 s, 3 s and 5 s (Δ*T*_1_ = 0.5*T*) and subjected to CWT/TOS-based analysis. Further experimental setting details can be found in [18].

#### (iv) Indicative results and discussion.

**Figure 4.**
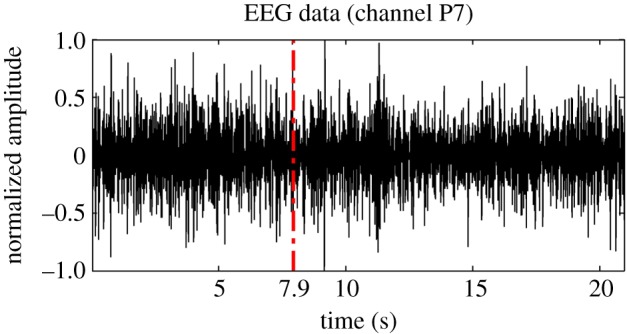
A characteristic example of EEG data from an experimental trial (channel P7). The time stamp of click1 appears at *t*_click1_ = 7.9 s of the experiment and it is denoted as the vertical dash-dotted line (adapted from Hadjileontiadis [18]).

**Figure 5.**
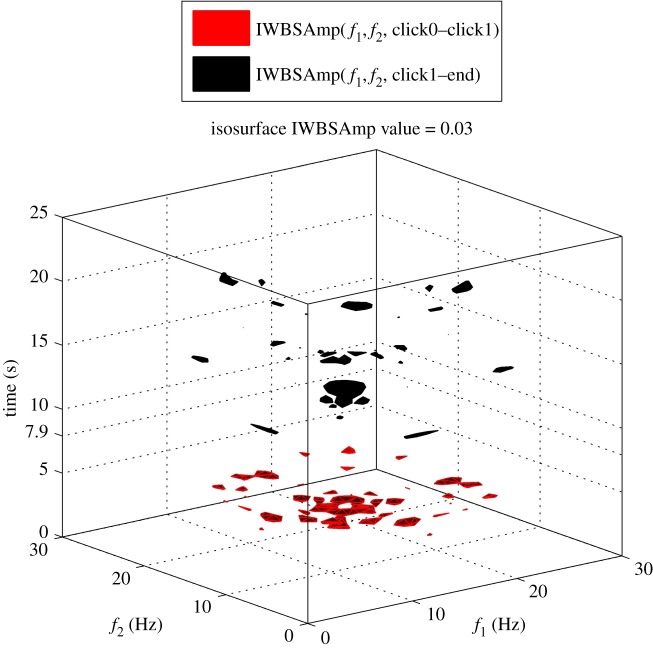
The estimated IWBSAmp corresponding to the EEG signal presented in figure 4, across its time duration in an isosurface fashion (isosurface value equals 0.03). Relax and pain periods defined before and after the borderline of click1, respectively, are colour coded, expressing an evident dynamic change in the bifrequency domain with time (adapted from Hadjileontiadis [18]).

An example of EEG recordings from one experimental trial from channel P7 is depicted in figure 4, whereas the corresponding IWBSAmp is shown in figure 5 in an isosurface fashion (the isosurface value was selected as 40% of the global maximum). In the latter, the evolution of the main bifrequency peaks before (red) and after (black) the occurrence of click1 at *t*_click1_ = 7.9 s (denoted also with a vertical dash-dotted line in figure 4) is displayed. Clearly, the specific experimental trial is characterized by QPC peaks that mainly lie at the range of *theta* up to *beta* bands. The transition between the latter seems to be affected by the initiation of the pain perception, as QPC peaks are starting to be less in number as we move from click1 towards the end of the experimental trial.

For the PCS#1, the classification results [18] have shown that the highest mean classification accuracy (CA) reaches 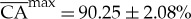, when *delta* and *beta* bands are both considered, all features of PCS#1 are used, the MAH classifier is employed and the NoR type of referencing is considered.

**Figure 6.**
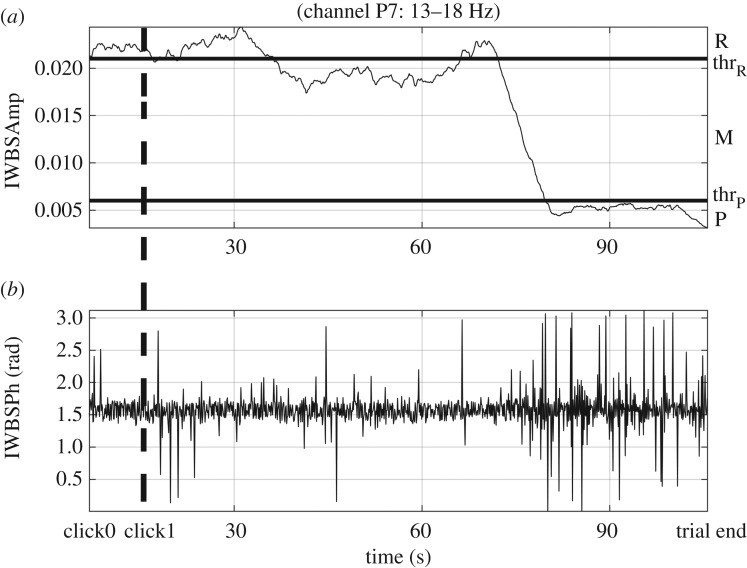
The estimated IWBSAmp (*a*) and IWBSPh (*b*) of EEG data acquired from channel P7 at *beta* frequency band from one subject, respectively, along with the thr_R,P_ defining the regions that correspond to the R/M/P states. The time stamp of click1 (within the time range of click0–trial end) is pointed out as a dashed vertical line (adapted from Hadjileontiadis [18]).

For the PCS#2, where dynamic pain characterization metrics are the point of interest, IWBSAmp and IWBSPh are considered as possible candidates. As a relevant paradigm, the estimated IWBSAmp (upper panel) and IWBSPh (lower panel) of EEG data acquired from channel P7 at *beta* frequency band from one subject are depicted in figure 6, respectively, along with the thr_R,P_ defining the regions that correspond to the no pain R, mild pain M, and severe pain P states. In addition, the *t*_click1_ (within the time range of click0–trial end) is pointed out as a dashed vertical line. From the IWBSAmp graph of figure 6*a*, it is clear that the subject activated click1 when he felt the first pain sensation ( ≈ 12 s), and then he tried to compensate that feeling up to ≈ 36 s, where he felt more pain, yet mild, sustaining his motivation to withstand pain up to ≈ 72 s (with a climax of his effort at ≈ 70 s), where he gradually started feeling more severe pain up to ≈ 79 s, which is sustained until the end of the trial. From the graph of figure 6*b*, the change in the variability of the estimated IWBSPh can be seen, with lower values when IWBSAmp >thr_R_, and higher ones when IWBSAmp <thr_P_. These changes in the IWBSPh variability, however, indicate more a qualitative rather than a quantitative measure, which supports the changes of the IWBSAmp (figure 6*a*) [18].

**Figure 7.**
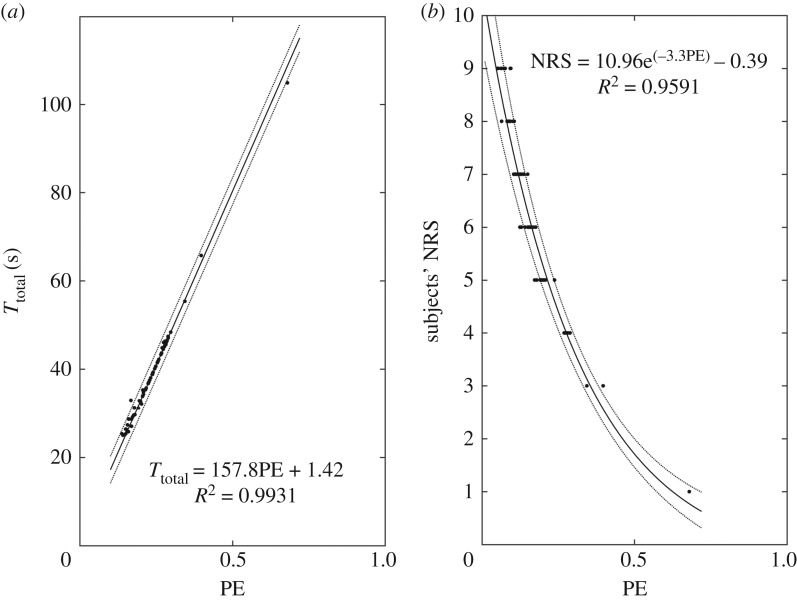
Comparison of PE with the subjects' *T*_total_ (*a*) and NRS (*b*) data. In both panels, the experimental data (dots) and the nonlinear least-squares fitting with a linear and exponential function (solid lines), respectively, along with the 95% confidence bounds (dashed lines) are depicted (adapted from Hadjileontiadis [18]).

Moreover, under the PCS#3, the issue of expressing, in a quantitative and objective way, the subjective judgement of pain intensity of each participant has been addressed via the proposed CWT/TOS-based PE parameter (see (3.3)). This is evident in figure 7, where the results from a comparison of PE with the subjects' *T*_total_ and NRS data are illustrated in figure 7*a*,*b*, respectively. In both panels, the experimental data (dots) were nonlinear least-squares fitted with a linear and exponential function (solid lines), respectively, with resulting coefficient of determination *R*^2^ > 0.9 and mean fitting error less than 1%. Moreover, the curves that correspond to the 95% confidence bounds are also depicted with dashed lines. As can be seen from figure 7*a*, there is a strong linear relationship between PE and *T*_total_ (*T*_total_ = 157.8PE + 1.42), showing that when the estimated PE increases the *T*_total_ also, analogously, increases. Moreover, based on figure 7*b*, there is an exponential relationship between PE and NRS (NRS = 10.96 e^(−3.3PE)^ − 0.39) reflecting an inverse analogy between PE and NRS; that is, the lower the subject's PE is, the higher the intensity of the perceived pain becomes; hence, the higher the marked NRS.

From the indicative results it is clear that, unlike previous approaches, the CWT/TOS-based feature space preserves the phase information by revealing the underlying QPC in a dynamic way. In addition, these features show the potential to more pragmatically reflect the EEG-based brain response and subjective pain characterization to the tonic cold pain stimulation. The observed changes in brain EEG response, captured by the CWT/TOS-based features during the evolution of the tonic cold pain, further indicates the potential for exploring brain dynamics associated with more pain types. Pain cases, such as chronic pain [37], and pain-related sensory and emotional processing in clinical populations can be considered. Clearly, there is potential for patients who are unable to communicate verbally, like sedated patients in the intensive care unit recovering from trauma and major surgery, as well as infant patients [38,39].

## 4. Concluding remarks

From the two case studies of the biomedical engineering field presented in the previous section, it is apparent that the CWT/TOS-based domain has the potential to efficiently handle the responses of real-life complex systems, such as breath sounds and EEG signals, in an effort to reveal underlying valuable information of the systems' functionality. The presented cases exemplify the transition from the data space (i.e. breath sounds and EEG) to an enhanced feature space, where the more pragmatic nature of the process is adopted, taking into consideration its non-Gaussianity, nonlinearity and non-stationarity. Building on the latter, the combination of CWT with TOS is an enhancement on traditional Fourier-based TOS representations, clearly providing an added value to the bifrequency domain expressed via: (a) the natural addition of a time axis and (b) the bispectral representation of transients, as wavelets have an inherent constant-*Q* filtering property well suited for their detection.

Furthermore, QPC detection based on CWT/TOS reduces time averages to a minimum, thus permitting short-lived, pulsed and intermittent events to be resolved. Relatively short data sequences are sufficient to perform an analysis, in contrast to the Fourier-based TOS that need long time series to obtain both sufficient frequency resolution and statistics. Estimates of the noise contribution and error level of the CWT/TOS parameters provide a criterion for the reliability of the relevant analysis results. Powerful noise reduction is an integral part of the standard technique as non-coherent contributions are averaged out, thus weak coherent signals can be detected in very noisy data. Moreover, the CWT/TOS parameters are more independent of the frame of reference and may be expected to be more useful in experiments where measurement in the local frame of reference is difficult.

A positive aspect of the combination of CWT with TOS in revealing coupling dynamics is that it introduces a relatively general approach for any system of coupled nonlinear oscillators where temporal data can be obtained. It is a highly relevant problem in numerous fields of research, e.g. cardiorespiratory interactions, brain oscillations (e.g. in exploring prediction of plausible seizures in epileptic patients during sleep), neuronal systems, electronic systems, coupled lasers, chemical reactions, to mention some. Hence, the presented combinatory approach provides a promising general-use tool for studying the nature and strength of coupling between two (or more) nonlinear oscillators whose basic frequencies change considerably in time [40].

Finally, the ways forward are open, considering that numerous applications and even hybrid approaches could be built on the CWT/TOS combination. A characteristic example would be to create multiple image sequences of CWT/TOS bifrequency representations across the time axis for feeding a deep learning network (e.g. convolutional neural network [41]). Consequently, instead of presenting to the latter original data from a system's output, more enriched, pragmatic and denoised representations could be provided by the CWT/TOS ones, setting the ground for new synergies between advanced signal processing and machine learning fields.

## Data accessibility

This article has no additional data.
